# Association Between Ultra-Processed Food Consumption and Lymphocyte Profile in People Living with HIV: A Cross-Sectional Study

**DOI:** 10.3390/medsci14030399

**Published:** 2026-07-17

**Authors:** Sofia Morais Tornis, David Michel de Oliveira, Fábio Morato de Oliveira, Luiz Rodrigo Augustemak de Lima, Mayara Bocchi, Eduardo Vignoto Fernandes

**Affiliations:** 1 Laboratory of Immunometabolism, Nutrition and Exercise (LABINE), Federal University of Jataí, Jataí 75801-615, Goiás, Brazileduardovignoto@ufj.edu.br (E.V.F.); 2 Laboratory of Human and Medical Genetics, Federal University of Jataí, Jataí 75801-615, Goiás, Brazil; 3 Institute of Physical Education and Sport, Federal University of Alagoas, Maceió 57072-970, Alagoas, Brazil

**Keywords:** immunosuppression, inflammation, immune system, processed food, dietary patterns, T lymphocytes

## Abstract

Introduction: Ultra-processed foods (UPFs) are characterized by high levels of additives such as sugars, fats, and preservatives and, due to their composition, represent a major public health concern, being associated with several adverse effects, including impacts on the immune system. People living with HIV (PLHIV) present immune dysfunctions resulting from viral infection and may therefore be particularly vulnerable to the effects of UPF consumption. In this context, investigating this association is necessary to support healthcare strategies and improve assistance for PLHIV. Objective: To investigate the relationship between UPF consumption and the lymphocyte profile of PLHIV. Methods: This was a cross-sectional study. PLHIV receiving outpatient follow-up care for at least six months participated in the study and were categorized according to UPF consumption (≤2 times/week; >2 times/week). Sociodemographic data, UPF consumption assessed through a food frequency questionnaire, anthropometric measurements, and blood samples were collected. Association, comparison, and correlation tests were performed, with statistical significance set at *p* < 0.05. After adjustment, lymphocyte percentage remained independently associated with UPF consumption. Results: A total of 92 PLHIV participated in the study, with a mean age of 43.0 ± 12.0 years. PLHIV with higher UPF consumption presented lower counts of CD3+ lymphocytes (*p* = 0.03), CD45+ lymphocytes (*p* = 0.01), and total lymphocytes (*p* = 0.03) compared to those with lower UPF consumption. UPF consumption was negatively correlated with CD3+ (r = −0.21; *p* = 0.04), CD4+ (r = −0.20; *p* = 0.05), CD45+ (r = −0.23; *p* = 0.02), and total lymphocyte counts (r = −0.25; *p* = 0.01). No associations were found between sociodemographic or clinical variables and UPF consumption among PLHIV (*p* > 0.05). Conclusions: High UPF consumption was associated with lower lymphocyte counts in PLHIV, suggesting a potential association between dietary patterns and immune status. Therefore, the implementation of public health policies and nutritional follow-up is necessary to reduce UPF consumption and promote healthy eating among PLHIV.

## 1. Introduction

Ultra-processed foods (UPFs) are industrial formulations composed of multiple processing steps and the addition of substances such as sugars, fats, salt, colorants, flavorings, and preservatives, aiming to increase product palatability and shelf life. Common examples include soft drinks, packaged snacks, stuffed cookies, instant noodles, and confectionery products [1].

The global increase in UPF consumption represents a major public health concern, as these foods contain compounds capable of disrupting the body’s antioxidant system, increasing inflammatory processes and favoring the production of reactive oxygen species, may negatively influence immune regulation and T-cell function [2]. These mechanisms, together with the increase in inflammatory biomarkers associated with UPF-rich diets, such as IL-6, TNF-α, leukocytes, and neutrophils, contribute to the development of chronic non-communicable diseases, including obesity, diabetes mellitus, hypertension, and dyslipidemia [2,3,4]. These conditions, in turn, impair immune system function by triggering exacerbated inflammatory processes and lymphocytic immunosuppression [2,3,4].

In people living with HIV (PLHIV), such immune dysfunction is intensified by the infection itself, since the human immunodeficiency virus (HIV) promotes chronic activation of the immune system by infecting CD4+ T lymphocytes and activating macrophages and cytotoxic lymphocytes (CD8+) [5]. However, this condition can be minimized with the appropriate use of medications that target proteins associated with viral replication, such as reverse transcriptase, integrase, and protease, thereby reducing the number of viral copies formed and circulating [6].

Beyond the benefits in controlling HIV infection, antiretroviral therapy (ART) may trigger adverse effects that contribute to immunometabolic imbalance, such as weight gain, hyperglycemia, increased lipid levels, and hypertension [7]. These alterations resemble those reported in individuals with high UPF consumption, suggesting that dietary habits may contribute to the metabolic and inflammatory disturbances observed in PLHIV receiving ART. Although not yet fully understood, part of these effects is hypothesized to result from similarities between the viral catalytic region and human proteins involved in lipid metabolism (namely cytoplasmic retinoic acid-binding protein type 1 and low-density lipoprotein receptor-related protein type 1) [8].

This context is particularly relevant due to the central role of T lymphocytes in the immune response. Among these cells, CD4+ T lymphocytes are responsible for coordinating immune responses through the activation of other immune system cells and the production of specific cytokines according to the pathogen to be fought [9]. Considering that UPF consumption has been associated with reduced number and function of T lymphocytes, and that HIV exhibits tropism for CD4+ T lymphocytes, it may be assumed that these factors act complementarily in compromising immunity. Thus, HIV infection compromises immune response coordination by targeting CD4+ T lymphocytes, resulting in progressive immune dysfunction, immunosuppression, and greater vulnerability to opportunistic infections [5].

In addition to the reduction in CD4+ T lymphocytes, other markers related to lymphocyte activation and functionality may also be altered in PLHIV. CD45+, a membrane phosphatase important for T-lymphocyte adhesion, migration, and activation, especially CD4+ cells, may show reduced levels in this population [10,11]. Similarly, lower levels of CD3+ proteins, components of the T-cell receptor complex and essential for immune signal transduction, have been described in individuals with high UPF consumption [12]. Nevertheless, little is known about the potential impact of UPF consumption on immunological parameters in people living with HIV, highlighting the need for further investigation in this population.

Therefore, considering the importance of nutritional status in maintaining immune function and in the clinical follow-up of PLHIV, investigating the possible association between UPF consumption and the lymphocyte profile of this population becomes relevant. In this context, the present study aimed to investigate the relationship between UPF consumption and the lymphocyte profile of PLHIV, seeking to broaden the understanding of possible associations between dietary habits and immunological parameters in this population.

## 2. Materials and Methods

### 2.1. Study Design and Data Collection Site

This study employed a cross-sectional design with a quantitative approach and was conducted in accordance with the recommendations of the STROBE (Strengthening the Reporting of Observational Studies in Epidemiology) guidelines for observational studies [13]. Data collection was carried out at the Testing and Counseling Center (TCA) and Specialized Care Service (SCS) of the municipality of Jataí, Goiás, Brazil, located at the Dr. Serafim de Carvalho State Hospital of Jataí (HEJ–Jataí), between January and November 2024.

### 2.2. Participant Recruitment, Sample Size Calculation, and Group Allocation

PLHIV were invited to participate in the study upon arrival at the TCA/SCS for routine blood sample collection. Participants were selected by convenience sampling according to the inclusion and exclusion criteria. Researchers approached PLHIV in the waiting room, and those interested in participating were invited to a private room for further clarification and signing of the Informed Consent Form.

Sample size was calculated using the G*Power^®^ software, version 3.1.9.2 (Institute of Experimental Psychology, Düsseldorf, Germany), with type I and type II errors defined as α = 0.05 and β = 0.05, respectively, to achieve an effect size of 0.80. Based on the sample size calculation, a minimum of 74 PLHIV was required. A total of 92 PLHIV participated in the study. PLHIV were allocated into two groups: PLHIV who consumed UPFs up to twice per week (*n* = 43) and PLHIV who consumed UPFs more than twice per week (*n* = 49).

The study was approved by the Research Ethics Committee of the Federal University of Jataí (approval number: 6390246; CAAE 71663823.2.0000.0187) on 5 October 2023, and all ethical principles of the Declaration of Helsinki were respected and followed. After approval by the Ethics Committee, the study was registered in the Brazilian Registry of Clinical Trials (ReBEC) under registration number RBR-10zr5mjp (https://ensaiosclinicos.gov.br/rg/RBR-10zr5mjp), accessed on 22 May 2024.

### 2.3. Inclusion and Exclusion Criteria

Participants were eligible if they were adults (≥18 years) of either sex with a confirmed diagnosis of HIV infection who had been receiving care at the Testing and Counseling Center/Specialized Care Service (TCA/SCS) in Jataí, Goiás, Brazil. Eligible participants were required to have been receiving continuous and uninterrupted antiretroviral therapy (ART) for at least six months prior to enrollment using the standard therapeutic regimen consisting of dolutegravir (50 mg), tenofovir (300 mg), and lamivudine (300 mg). This eligibility criterion was adopted to ensure greater clinical and immunological stability and to minimize the influence of recent ART initiation on the evaluated immunological parameters.

Individuals were excluded if they were younger than 18 years of age, pregnant or breastfeeding, had not been receiving continuous ART for at least six months, failed to complete all study procedures, or withdrew from the study before its completion.

### 2.4. Procedures

Sociodemographic information (education level, marital status, age, and sex), lifestyle habits (alcohol and tobacco consumption), and clinical information (duration of infection and ART use), as well as physical activity level (PAL), dietary intake assessment, blood collection, and anthropometric evaluation were collected from the PLHIV.

#### 2.4.1. Physical Activity Level (PAL)

PAL was assessed using the International Physical Activity Questionnaire–Short Form (IPAQ-SF). The IPAQ-SF classifies participants as very active, active, insufficiently active, or sedentary [14]. For data presentation purposes in the present study, individuals were classified as active (very active + active) or inactive (insufficiently active + sedentary), as proposed by Oliveira et al. [15].

#### 2.4.2. Dietary Intake Assessment

Dietary intake was assessed using a questionnaire according to the degree and nature of food processing [16]. Examples of foods were classified into: i. unprocessed or minimally processed foods; ii. oils, fats, salt, and sugar; iii. processed foods; and iv. ultra-processed foods. Participants reported their frequency of consumption. This study considered only UPFs.

To estimate UPF consumption frequency, PLHIV were asked about the consumption of several types of cookies, ice cream, candies and sweets in general, sugary breakfast cereals, cakes and cake mixes, cereal bars, soups, instant noodles and seasonings, sauces, packaged snacks, soft drinks and other sugary beverages, sweetened and flavored yogurts and dairy drinks, energy drinks, frozen and ready-to-heat products such as pasta, pizzas, hamburgers, breaded chicken or fish, as well as processed meat products such as nuggets, sausages, and other processed meats, sliced bread, hamburger or hot dog buns, sweet breads, and bakery products containing ingredients such as hydrogenated vegetable fat, starch, whey protein, emulsifiers, and other additives.

During the interview, after PLHIV reported the frequency of UPF consumption, researchers recorded the information and converted daily consumption frequency as follows: every day = 1; 5–6 times/week = 0.79; 2–4 times/week = 0.43; once/week = 0.14; 1–3 times/month = 0.07; and never or almost never = 0 [17].

#### 2.4.3. Blood Collection and Hematological Examinations

Participants were instructed to fast for 8–12 h prior to blood collection. Venous blood samples were collected from the antecubital fossa via venipuncture of the median cubital, cephalic, or basilic vein using a vacuum blood collection system (Vacutainer^®^, Becton Dickinson, Franklin Lakes, NJ, USA). Blood samples were collected into EDTA-containing tubes (BD Vacutainer^®^, Becton Dickinson, Franklin Lakes, NJ, USA) and immediately transported to the laboratory for hematological, immunological, and virological analyses. After collection, blood samples were processed for laboratory analyses, including complete blood count, CD3+, CD4+, CD8+, and CD45+ cell counts, as well as plasma HIV-1 viral load determination.

Complete blood count parameters (red blood cells, hemoglobin, hematocrit, lymphocytes, leukocytes, neutrophils, monocytes, and platelets) were analyzed using the Sysmex XN-350 automated hematology analyzer (Sysmex, Kobe, Japan) with whole blood samples. CD3+, CD4+, CD8+, and CD45+ cell counts were determined by flow cytometry using the BD FACSVia™ system with BD Multitest™ reagents. Plasma HIV-1 viral load was quantified using the cobas^®^ 5800 system (Roche Diagnostics, Indianapolis, IN, USA) by real-time polymerase chain reaction (RT-PCR).

#### 2.4.4. Anthropometric Assessment and Body Composition

Body weight was measured using a calibrated analog scale (Welmy^®^, Welmy Indústria e Comércio Ltda., Santa Bárbara d’Oeste, SP, Brazil) with 100 g precision. Height was measured using a stadiometer coupled to the scale, with 0.5 cm precision. Body mass index (BMI) was calculated by dividing body weight (kg) by height squared (m^2^). Waist and hip circumferences were measured using a (Sanny^®^, American Medical do Brasil Ltda., São Bernardo do Campo, SP, Brazil) anthropometric tape, and the waist-to-hip ratio (WHR) was subsequently calculated.

### 2.5. Bias Control

To minimize bias, all participants were recruited according to standardized inclusion and exclusion criteria. Interviews were conducted by previously trained researchers using validated instruments. Anthropometric and laboratory assessments followed standardized protocols, and the equipment used was regularly calibrated.

### 2.6. Missing Data Handling

There was no missing data for the main analyzed variables. Participants who did not complete all study procedures were excluded from the analyses.

### 2.7. Statistical Analysis

Initially, data distribution was assessed using the Kolmogorov–Smirnov test. As most continuous variables did not meet the assumption of normality, results were expressed as median and interquartile range (IQR; 25th–75th percentile), whereas categorical variables were presented as absolute frequencies and percentages. Comparisons between groups were performed using the Mann–Whitney U test for continuous variables and Fisher’s exact test for categorical variables, with Odds Ratios (ORs) and 95% confidence intervals (95% CIs) calculated when appropriate. Associations between ultra-processed food consumption and clinical, anthropometric, and immunological variables were evaluated using Spearman’s rank correlation coefficient (ρ). Statistical significance was established at *p* < 0.05. Additionally, a multiple linear regression model (least squares method) was performed to identify independent predictors of ultra-processed food consumption. Variables with *p* < 0.20 in the bivariate analyses were selected for inclusion in the matrix. To avoid multicollinearity bias, Variance Inflation Factor (VIF) analysis was performed, and variables with VIF > 10 were excluded from the model. Model fit quality was evaluated using the multiple correlation coefficient (R) and determination coefficients (R^2^ and adjusted R^2^). Statistical analyses were performed using GraphPad Prism 9.5.1, and the minimum significance level adopted was *p* < 0.05.

## 3. Results

A total of 92 people living with HIV (PLHIV) were included in the study. Participants had a mean age of 43.0 ± 12.0 years, body weight of 73.8 ± 18.5 kg, height of 1.66 ± 0.08 m, and body mass index (BMI) of 26.5 ± 6.3 kg/m^2^. The mean waist circumference was 89.4 ± 15.4 cm, hip circumference was 98.9 ± 18.0 cm, and waist-to-hip ratio (WHR) was 0.92 ± 0.16. The mean time since HIV diagnosis was 113.8 ± 72.1 months, while the mean duration of antiretroviral therapy (ART) was 96.8 ± 61.5 months. Table 1 presents the sociodemographic profile of the PLHIV participating in the study. A tendency toward higher UPF consumption was observed among men (60.3% in the ≥2 times/week group). In addition, most PLHIV, regardless of UPF consumption frequency, were single or without partners, had lower educational attainment, were non-smokers, reported low alcohol consumption, and were physically active.

Table 2 presents the median and interquartile range values of the immunobiochemical and nutritional parameters obtained from blood analysis of PLHIV according to UPF consumption frequency. It was observed that patients with higher UPF consumption (≥2 times/week) presented lower values of CD3+ cells (1596 vs. 1365 cells/mm^3^, *p* = 0.03), CD45+ cells (2146 vs. 1918 cells/mm^3^, *p* = 0.01), and percentage of total lymphocytes (33.5% vs. 28%, *p* = 0.03), indicating a lower number of circulating lymphocytes.

Regarding CD4+ T lymphocytes, CD8+ T lymphocytes, CD4+/CD8+ ratio, neutrophils, red blood cell count, hemoglobin, hematocrit, total leukocytes, platelets, and monocytes, no differences were observed between the groups (*p* > 0.05).

The multivariate linear regression analysis showed a significant coefficient of determination (R^2^ = 0.151; *p* = 0.01), with no independent association observed between UPF consumption and age, sex, educational level, BMI, or PAL (Table 3). Absolute counts of CD3+, CD4+, and CD8+ T lymphocytes, the CD4/CD8 ratio, and CD45+ levels were not included in the matrix due to collinearity with lymphocyte percentage. Similarly, waist and hip circumferences, as well as WHR, were excluded due to collinearity with BMI.

The adjusted multivariate analysis revealed associations only between UPF consumption and duration of ART use (β = 0.001; *p* = 0.01), as well as lymphocyte percentage (β = −0.007; *p* = 0.03).

**Figure 1:**
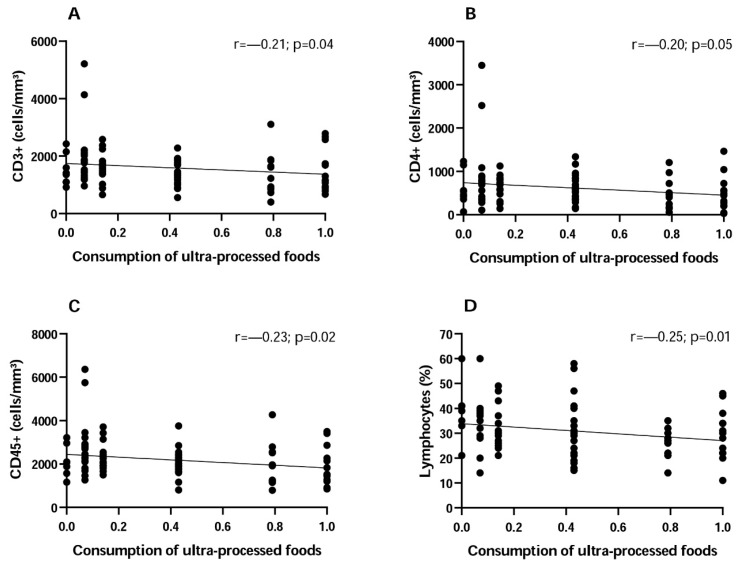
Correlation between daily UPF consumption and CD3+ (**A**), CD4+ (**B**), CD45+ (**C**), and lymphocyte percentage (**D**) in PLHIV.

Figure 1 illustrates the correlations between ultra-processed food (UPF) consumption and immunological markers in PLHIV. Significant inverse correlations were observed between UPF consumption and CD3+ T-cell counts (r = −0.21, *p* = 0.04), CD4+ T-cell counts (r = −0.20, *p* = 0.05), CD45+ cell counts (r = −0.23, *p* = 0.02), and total lymphocyte percentage (r = −0.25, *p* = 0.01). No significant correlations were identified between UPF consumption and the remaining immunological, hematological, or anthropometric variables evaluated (all *p* > 0.05).

## 4. Discussion

The findings of the present study indicate that higher UPF consumption is associated with alterations in the immunological profile of PLHIV, characterized by lower counts of specific lymphocyte subpopulations. These results suggest that greater consumption of ultra-processed foods may be associated with subtle but potentially relevant alterations in the immunological profile of PLHIV [3]. Thus, the present study expands current knowledge regarding the association between nutrition and immune function in a clinically vulnerable group.

Importantly, plasma HIV-1 viral load did not differ significantly between the study groups, indicating comparable virological status regardless of ultra-processed food consumption. This finding reduces the likelihood that the observed differences in lymphocyte counts were primarily explained by differences in viral replication. Although viral suppression does not eliminate the chronic immune activation characteristic of HIV infection, the comparable virological status between groups strengthens the interpretation that the associations observed are unlikely to be attributable to differences in HIV disease control. Nevertheless, residual confounding cannot be completely excluded given the observational and cross-sectional nature of the study.

Regarding the immunological profile, individuals with higher UPF consumption showed reduced counts of CD3+ lymphocytes, CD45+ lymphocytes, and lower percentages of total lymphocytes. These findings indicate a correlation between higher UPF consumption and decreased lymphocyte populations, in agreement with the literature describing the deleterious effects of UPF-rich diets on nutritional quality, inflammatory status, and immunometabolic health [7]. Similar findings have been described in clinical studies involving PLHIV, in which the consumption of foods with poor nutritional quality, such as UPFs, was associated with a worse lymphocyte profile, including reductions in T-cell populations and alterations in the CD4/CD8 ratio, even among individuals receiving effective ART [18].

Previous studies have shown that compounds formed during industrial food processing, such as acrylamide, acrolein, advanced glycation end products (AGEs), and polycyclic aromatic hydrocarbons, promote increased oxidative stress and activate pro-inflammatory pathways in the human body, elevating inflammatory markers such as IL-6, TNF-α, leukocytes, and circulating neutrophils [4]. Furthermore, contaminants from UPF packaging, such as bisphenols and microplastics, may further contribute to immune system impairment [4]. These mechanisms may explain the findings observed in the group consuming ultra-processed foods more than twice per week, which showed a tendency toward neutrophilia (a cell type associated with inflammatory responses), since the chronic inflammation already present in this group may become even more pronounced due to the amount of UPFs consumed [3].

This scenario is particularly relevant in PLHIV because HIV exhibits tropism for CD4+ T lymphocytes, resulting in their progressive depletion and impaired coordination of immune responses. In addition, HIV infection is associated with persistent systemic inflammation driven by chronic immune activation, intestinal barrier disruption, and increased microbial translocation into the systemic circulation [5]. Consequently, PLHIV may be especially susceptible to additional inflammatory and immunological challenges associated with environmental and lifestyle factors, including dietary patterns [4]. These processes contribute to increased oxidative stress and metabolic and immunological alterations, which may influence T-lymphocyte homeostasis [3]. Thus, when combined with a dietary pattern based on UPFs, characterized by low intake of fiber, antioxidants, and bioactive compounds, these mechanisms tend to intensify, further amplifying inflammation and deepening immune dysfunction in PLHIV [3,4].

Even under ART, inflammation persists and is evidenced by reductions in lymphocyte markers, particularly CD4+ T lymphocytes. This condition increases vulnerability to additional inflammatory stimuli, such as those induced by high UPF intake [4]. Moreover, additives such as emulsifiers, artificial sweeteners, nitrates, colorants, and stabilizers are associated with alterations in microbiota and increased intestinal permeability, which may amplify microbial translocation, a mechanism already exacerbated by HIV infection [4].

Although the present study focused on PLHIV, similar mechanisms may also occur in healthy individuals. Diets characterized by high consumption of ultra-processed foods have been associated with low-grade systemic inflammation, oxidative stress, alterations in gut microbiota composition, and metabolic disturbances, all of which may influence immune homeostasis [19]. However, because healthy individuals generally have preserved immune function and do not experience the chronic immune activation associated with HIV infection, the magnitude and clinical consequences of these dietary effects are likely to be less pronounced than those observed in PLHIV [3,4,19].

Based on these findings, it may be suggested that higher UPF consumption is associated with alterations in the cellular immune profile of PLHIV, as confirmed by the negative correlations observed between UPF consumption and the immunological markers CD3+, CD4+, CD45+, and total lymphocyte percentage, both in the present study and in previous investigations [20]. The concomitant reduction in CD3+ and CD45+ reflects lower circulating lymphocyte counts; however, the present study did not assess functional or phenotypic markers of T-cell activation. Therefore, no conclusions regarding lymphocyte activation or signaling can be drawn from these findings. Likewise, the lower CD4+ counts observed among participants with higher UPF consumption may indicate alterations in immune status, although the underlying biological mechanisms remain to be elucidated [5]. These findings suggest that the impact of UPF consumption in PLHIV may be associated with changes in the cellular immune profile, although mechanistic studies are needed to clarify these associations.

Similarly, reduced CD4+ levels are particularly relevant in PLHIV, since CD4+ count is one of the main clinical markers of HIV disease progression because these cells are the primary target of HIV infection [21]. The reduction in total lymphocyte counts observed among individuals with higher UPF consumption may reflect a less favorable immunological profile in PLHIV. However, given the observational design of the study, these findings should not be interpreted as direct evidence of immunosuppression [5,21]. Although the absolute differences in lymphocyte counts observed between the groups were relatively modest, they may still be physiologically relevant in PLHIV. Because this population is characterized by chronic immune activation and persistent immune dysregulation, even subtle alterations in lymphocyte populations may reflect changes in immune homeostasis. Nevertheless, these findings should be interpreted with caution, and their clinical significance should be confirmed in prospective studies evaluating clinical outcomes and functional immune parameters.

To further evaluate the observed associations, a multivariate linear regression model was performed. The analysis demonstrated that the inverse association between ultra-processed food consumption and total lymphocyte percentage remained statistically significant after adjustment for age, sex, educational level, and BMI. These findings suggest that the observed relationship remained independent of the demographic, anthropometric, and educational variables included in the model. However, because more comprehensive socioeconomic information was not collected, the potential influence of socioeconomic status on the observed associations cannot be completely excluded. This finding is important because it suggests that the impact of an ultra-processed diet on immune homeostasis in PLHIV is not merely a reflection of anthropometric nutritional status or demographic characteristics, but rather an independent association between dietary quality and lymphocyte populations.

In addition to the immunological profile, duration of ART exposure emerged as an independent and positive predictor of UPF consumption. This result indicates that chronicity of treatment is associated with a progressive increase in UPF intake. The observed association suggests that prolonged ART use correlates with the adoption of dietary patterns based on highly convenient and palatable foods. Therefore, these data demonstrate that treatment duration is a clinical determinant of dietary quality, reinforcing the need for continuous nutritional monitoring as an integral component of PLHIV management protocols.

Taken together, these findings support the hypothesis that dietary patterns may be associated with immune modulation in PLHIV [18]. The results of the present study contribute to a growing body of evidence highlighting the potential role of nutrition in the immunometabolic health of this population. Furthermore, they suggest that nutritional strategies aimed at reducing ultra-processed food consumption and promoting diets rich in fruits, vegetables, and other minimally processed foods may represent valuable complementary approaches in the clinical management of PLHIV. Nevertheless, given the cross-sectional nature of the study, causal relationships between UPF consumption and the observed immunological alterations cannot be established. In addition, the clinical heterogeneity of the sample, including differences in time since HIV diagnosis, duration of ART, and individual characteristics related to health status and lifestyle, may have influenced the observed results. Although educational level was included in the multivariate analysis, more comprehensive socioeconomic information, such as household income and employment status, was not collected. Because socioeconomic factors may influence dietary habits, access to healthy foods, and health-related behaviors, their potential confounding effect on the observed associations cannot be completely excluded. Therefore, future longitudinal and interventional studies should incorporate broader socioeconomic indicators and evaluate, over time, the interaction between dietary patterns, infection progression, ART response, and the evolution of immunological markers, as well as their impacts on metabolic health and quality of life in PLHIV.

Importantly, the present study has important strengths, particularly the simultaneous evaluation of different immunological markers (CD3+, CD4+, and CD45+), allowing for a broader and more integrated analysis of cellular immune response in PLHIV. Furthermore, by investigating a modifiable factor such as dietary pattern, the study enhances its clinical relevance and reinforces the potential of non-pharmacological interventions in the care of this population. Finally, the integration of nutritional and immunological aspects in a vulnerable group contributes consistently to advancing knowledge regarding the relationship between nutrition and immunity in PLHIV.

## 5. Conclusions

The findings of this study suggest that frequent UPF consumption is associated with alterations in the immunological profile of PLHIV, particularly involving lymphocytes. Individuals with higher intake of ultra-processed foods showed reductions in CD3+ and CD45+ lymphocyte counts and in total lymphocyte percentage, with a tendency toward decreased CD4+ and CD8+ levels. These results suggest that a dietary pattern characterized by high consumption of industrialized products rich in sugars, fats, and additives and poor in essential nutrients may be associated with alterations in the immunological profile of an immunocompromised population.

In this context, promoting dietary patterns based on unprocessed and minimally processed foods while reducing the consumption of ultra-processed foods may represent an important complementary strategy for supporting immune health and improving quality of life among PLHIV. Finally, longitudinal studies are recommended to further investigate the mechanisms underlying the observed associations and to evaluate the impact of dietary modifications on immune function and nutritional status in this population. Such studies are necessary to confirm the findings of the present study, support evidence-based nutritional intervention strategies, and contribute to improving health promotion actions directed toward PLHIV, particularly regarding immunological health and quality of life.

## Figures and Tables

**Table 1:** Association between the frequency of ultra-processed food consumption among PLHIV and sociodemographic variables and lifestyle habits.

Categorical Variables	Daily Consumption of Ultra-Processed Foods		
	<2 Times/Week (*n* = 43)	≥2 Times/Week (*n* = 49)	*p*
	*n* (%)	*n* (%)	
Sex			
Female	21 (48.8)	15 (41.7)	0.08
Male	22 (39.2)	34 (60.3)	
Marital status			
With partner	15 (34.9)	18 (36.7)	0.99
Without partner	28 (65.1)	31 (63.3)	
Educational level			
≤9 years	27 (62.8)	35 (71.4)	0.50
>9 years	16 (37.3)	14 (28.6)	
Smoking status			
Yes	13 (30.2)	20 (40.8)	0.38
No	30 (69.8)	29 (59.2)	
Alcohol consumption			
Yes	19 (44.2)	26 (53.1)	0.41
No	24 (55.8)	23 (46.9)	
PAL			
Active	29 (67.4)	36 (73.5)	0.67
Inactive	14 (32.6)	13 (26.5)	

Legend: PLHIV, people living with HIV; PAL, physical activity level.

**Table 2:** Immunobiochemical and nutritional parameter profile of PLHIV.

Categorical Variables	Daily Consumption of Ultra-Processed Foods		*p*
	<2 Times/Week (*n* = 43)	≥2 Times/Week (*n* = 49)	
Viral load (copies/mL)	0 (0–38)	0 (0–61)	0.17
CD3+ (mm^3^)	1596 (1328–2093)	1365 (957–1833)	0.03
CD4+ (mm^3^)	693 (357–822)	485 (279–754)	0.11
CD8+ (mm^3^)	879 (604–1264)	761 (519–972)	0.07
CD4+/CD8+	0.69 (0.48–1.06)	0.68 (0.36–1.18)	0.45
CD45+ (mm^3^)	2146 (1808–2875)	1918 (1478–2346)	0.01
Lymphocytes (%)	33.5 (26.7–39.0)	28.0 (22.0–34.0)	0.03
Red blood cells (million/mm^3^)	4.7 (4.3–5.0)	4.7 (4.4–5.0)	0.97
Hemoglobin (g/dL)	14.3 (13.1–15.0)	14.3 (13.5–15.4)	0.72
Hematoocrit (%)	41.1 (39.2–43.6)	40.9 (39.0–43.9)	0.91
Leukocytes (mm^3^)	5950 (4570–6953)	5780 (4620–7540)	0.85
Neutrophils (%)	58.0 (50.5–63.2)	62.0 (54.0–69.0)	0.06
Monocytes (%)	7 (5–8)	7 (6–8)	0.76
Platelets (Thousand/mm^3^)	217 (174–269)	220 (191–262)	0.96

Legend: CD, cluster of differentiation. Values are presented as median and interquartile range (25th–75th percentile). The Mann–Whitney test was used for group comparisons, with a significance level set at *p* < 0.05.

**Table 3:** Multivariate Linear Regression of Factors Associated with Ultra-Processed Food Consumption in PLHIV.

	Multivariate Linear Regression Analysis				
Categorical Variables	Coef β	SE	CI 95%	*p*	VIF
Constant	0.838	0.320	0.200 to 1.47	0.01	-
Age	−0.000	0.003	−0.007 to 0.007	0.96	1.56
Sex	−0.070	0.085	−0.240 to 0.099	0.40	1.25
Educational level	0.003	0.026	−0.048 to 0.055	0.90	1.61
BMI	−0.009	0.006	−0.023 to 0.003	0.14	1.09
PAL	0.015	0.080	−0.146 to 0.176	0.85	1.02
ART	0.001	0.000	0.000 to 0.002	0.01	1.21
Lymphocytes	−0.007	0.003	−0.015 to −0.000	0.03	1.08
		*R*^2^ = 0.151	*R*^2^ adjusted = 0.074		

Legend: Coef β: Beta coefficient; SE: Standard Error; 95% CI: 95% Confidence Interval; VIF: Variance Inflation Factor; PAL: Physical Activity Level; ART: Antiretroviral Therapy; BMI: Body Mass Index.

## Data Availability

The original contributions presented in this study are included in the article. Further inquiries can be directed to the corresponding author.

## Abbreviations

The following abbreviations are used in this manuscript:

95% CI	95% confidence interval
AGEs	Advanced glycation end products
ART	Antiretroviral therapy
BMI	Body mass index
CAAE	Certificate of Presentation for Ethical Consideration
CD3	Cluster of differentiation 3
CD4	Cluster of differentiation 4
CD8	Cluster of differentiation 8
CD45	Cluster of differentiation 45
CEP	Research Ethics Committee
EDTA	Ethylenediaminetetraacetic acid
HEJ	Dr. Serafim de Carvalho State Hospital of Jataí
HIV	Human immunodeficiency virus
IL-6	Interleukin 6
IPAQ-SF	International Physical Activity Questionnaire—Short Form
IQR	Interquartile range
LABINE	Laboratory of Immunometabolism, Nutrition and Exercise
OR	Odds ratio
PAL	Physical activity level
PLHIV	People living with human immunodeficiency virus
ReBEC	Brazilian Registry of Clinical Trials
SCS	Specialized Care Service
STROBE	Strengthening the Reporting of Observational Studies in Epidemiology
TCA	Testing and Counseling Center
TNF-α	Tumor necrosis factor alpha
UPF	Ultra-processed food
UPFs	Ultra-processed foods
VIF	Variance inflation factor
WHR	Waist-to-hip ratio
